# Scanning accuracy of an intraoral scanner according to different inlay preparation designs

**DOI:** 10.1186/s12903-023-03233-2

**Published:** 2023-07-24

**Authors:** Yeri Park, Jae-Hoon Kim, Jeong-Kil Park, Sung-Ae Son

**Affiliations:** 1 Department of Conservative Dentistry, School of Dentistry, Pusan National University, Yangsan, Republic of Korea; 2 Department of Dental Education, Dental and Life Science Institute, School of Dentistry, Pusan National University, Dental Research Institute, Yangsan, Republic of Korea; 3 Department of Conservative Dentistry, Dental and Life Science Institute, School of Dentistry, Pusan National University, Dental Research Institute, Geumo-Ro 20, Mulgeum-Eup, Box 50612, Yangsan, Republic of Korea

**Keywords:** Inlay designs, Intraoral scanner, CAD/CAM, Accuracy, Trueness, Precision

## Abstract

**Background:**

The accuracy of intraoral scanning plays a crucial role in the workflow of computer-assisted design/computer-assisted manufacturing. However, data regarding scanning accuracy for inlay preparation designs are lacking. The purpose of this in vitro study was to evaluate the influence of the depth of the occlusal cavity and width of the gingival floor of the proximal box on the trueness and precision of intraoral scans for inlay restoration.

**Methods:**

Artificial teeth were used in this study. Four types of preparations for mesio-occlusal inlay were performed on each #36 artificial tooth depending on two different depths of the occlusal cavity (1 mm and 2 mm) and widths of the gingival floor of the proximal box (1.5 mm and 2.5 mm). Artificial teeth were scanned 10 times each with Cerec Primescan AC, and another scan was performed subsequently with a laboratory scanner as a reference (*n* = 10). Standard tessellation language files were analyzed using a three-dimensional analysis software program. Experimental data were analyzed using two-way analysis of variance and the Bonferroni multiple comparison test.

**Results:**

The narrow shallow group had significantly higher deviation values for trueness than the wide deep group (*p* < 0.05). The wide deep group had the lowest average deviation value for trueness and there was no significant difference between the narrow deep and wide shallow groups (*p* > 0.05). For the mean maximum positive deviation, the wide groups had significantly lower values than the narrow groups (*p* < 0.05). Trueness was affected by both the width and depth(*p* < 0.05), whereas the mean maximum positive deviation was affected by the width (*p* < 0.05). The mean maximum negative deviation was affected by all three factors (*p* < 0.05). Precision was affected by the depth and the interaction between the depth of the occlusal cavity and width of the gingival floor (*p* < 0.05).

**Conclusions:**

The design of different inlay cavity configurations affected the accuracy of the digital intraoral scanner. The highest average deviation for trueness was observed in the narrow shallow group and the lowest in the wide deep group. With regard to precision, the narrow shallow group showed the lowest average deviation, and the narrow deep group showed highest value.

## Background

Intraoral digital scanning and computer-assisted design/computer-assisted manufacturing (CAD/CAM) have become popular and have been used as alternatives to conventional impression-making and -casting methods, especially with the introduction of a new scanner design [1]. Conventional impression procedures involve several different steps, and every step bears the possibility of introducing errors, thereby negatively influencing the quality of the final model [2]. Digital intraoral impressions eliminate many steps, as the original tooth surface can be scanned in a single step [2]. In addition, intraoral scanner (IOS) systems have other advantages including time efficiency, increased patient comfort, and data fusion options within the CAD/CAM workflow [3].

The accuracy of digital scanning is a major factor in successful inlay restorations [2]. The International Organization for Standardization (ISO) defined the accuracy of measurements as consisting of two components—trueness and precision—to standardize the terminology for evaluating data of digital image scans (ISO-5725–1) [4–6]. Trueness indicates how much deviation from the original surface is present in the extraoral or digital mode. In contrast, precision refers to the proximity between test results when a certain scanner repeatedly takes images of a specific subject, and is an index that indicates the difference between images obtained by repeatedly scanning under the same conditions [5, 7–9]. These two factors describe the IOS accuracy together [2, 5, 7].

Inlays for a Class II cavity including a proximal box have a more complex configuration than those for a Class I cavity or supragingival simple crowns. These designs complicate the clinical steps of cavity preparation, optical scanning, margin readings, adhesive cementation, finishing, and polishing [5, 7]. The quality of the intraoral scan data may vary depending on the accessibility of the IOS and the range of access. In addition, the cavity type can affect the access of and the scattering of light from the IOS. Moreover, the quality of the image generated with an IOS varies depending on the location of the gingival margin of the proximal box [4, 10]. In addition, studies have considered the IOS accuracy of inlay cavity designs according to the tooth location and distance or presence of the adjacent tooth [4, 11–13]. Previous studies show consistent result that proximal box negatively affects the accuracy when scanning is performed in the oral cavity [4, 12, 14]. The depth of the proximal box cavity can be adversely affected by reflection and distortion of the scanner due to the influence of the surrounding teeth and soft tissues of the gingiva. Changes in morphology of the proximal box can interrupt the scanning light rayed in parallel, which makes it difficult for the accurate scanning. For CAD-CAM based inlay restoration, several studies reported that the effect of the cavity design such as bucco-lingual width and depth of proximal box have an effect on the trueness and precision of digital scan data [4, 12]. However, clinical guidelines for obtaining precise scan data for inlay restorations according to the cavity design remain lacking [4]. This study aimed to evaluate the influence of the depth of the occlusal cavity and width of the gingival floor of the proximal box on the trueness and precision of a digital IOS for inlay cavities. The null hypothesis was that the depth of the occlusal cavity and width of the gingival floor of the proximal box would not affect the accuracy of the digital scan data.

## Methods

### Preparation designs of artificial teeth

**Fig. 1:**
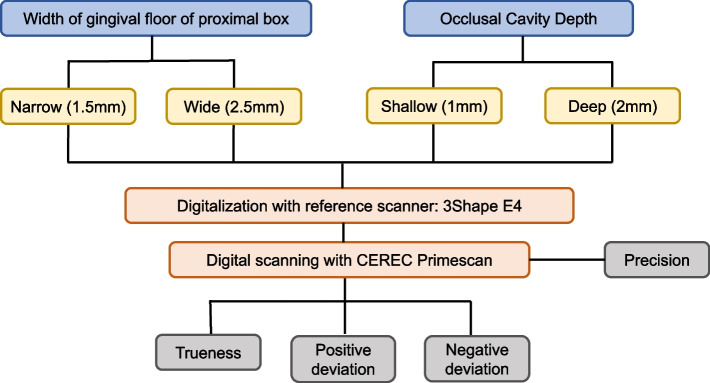
Schematic workflow of the study

**Fig. 2:**
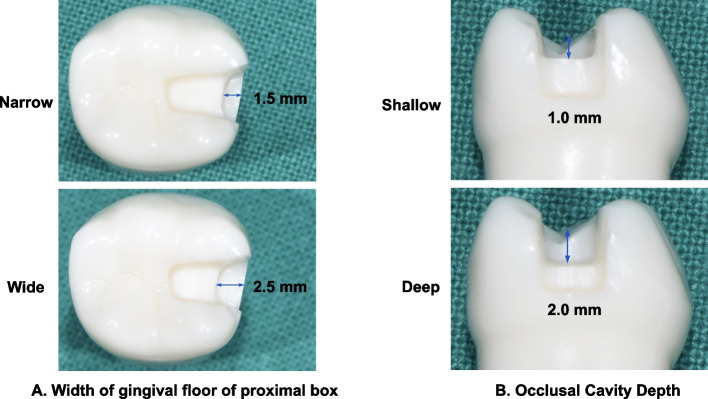
Preparation designs of teeth according to the width of the gingival floor of the proximal box and occlusal cavity depth

Figure 1 shows the overall workflow of this study. Four artificial mandibular left first molars (A5AN-500; Nissin Dental) were prepared under a microscope (M320 F12, Leica Instruments) using inlay preparation rotary instruments (845KR.314.016; Komet Dental) by one operator. Four cavity designs were prepared on the basis of the width of the gingival floor of the mesio-occlusal (MO) proximal box (1.5 mm, 2.5 mm) and depth of the occlusal cavity (1 mm, 2 mm) of the proximal box. The isthmus and the depth of the gingival floor of the proximal box were fixed. Table 1 presents the description of the cavity design form for each group in this study, and Fig. 2 shows the occlusal and proximal views of the tooth preparation designs according to the mesio-distal width of the gingival floor of the proximal box and depth of the occlusal cavity.

**Table 1:** Description of cavity preparation designs as per groups

Group	Cavity form
Narrow shallow	MO cavity with 1.5 mm width of gingival floor of proximal box and 1.0 mm occlusal cavity depth
Narrow deep	MO cavity with 1.5 mm width of gingival floor of proximal box and 2.0 mm occlusal cavity depth
Wide shallow	MO cavity with 2.5 mm width of gingival floor of proximal box and 1.0 mm occlusal cavity depth
Wide deep	MO cavity with 2.5 mm width of gingival floor of proximal box and 2.0 mm occlusal cavity depth

*MO* Mesio-occlusal

### Digital scanning procedure

Control scan data were obtained using a reference scanner (3Shape E3; 3Shape A/S), which converts scan files into the standard tessellation language (STL) format. Digital scans were performed with an IOS (CEREC Primescan AC v. 5.1.0; Dentsply Sirona). The scanning procedure was repeated 10 times for each group, according to the manufacturer’s instructions by the same operator. The acquired scan data were exported as raw STL files, without smoothing.

### Data superimposition and image analysis

A three-dimensional (3D) inspection software program (GOM Inspect 2018; GmbH) was used to evaluate the accuracy of the scan data for the experimental models. From the obtained STL files, only the MO inlay cavity part of the mandibular first molars were used for data analysis, and parts of the tooth that were not related to our study were removed from the software program. In order to measure the deviation values for trueness, which represents the deviation between the STL file obtained from Primescan and the original STL file obtained from the reference scanner, each cavity design was obtained by superimposing the reference data and STL files of the experimental model using the initial alignment and 3D best-fit alignment methods. All scanned data values are shown using the average deviation. When overlapping between STL files for trueness measurement, the average maximum positive and negative deviations were automatically calculated with the GOM inspect software program and were measured as local deviation values. In order to evaluate the precision values between the repeatedly acquired intraoral scanner images, the STL files of each experimental group were overlapped with each of other obtained scan data within the same group. This procedure was performed in the same manner using the initial alignment and 3D best-fit alignment methods (*n* = 45). A color difference map was created by the qualitative analysis of the reference and experimental data using the visual images.

### Statistical analysis

Experimental data were analyzed using a statistical software program (IBM SPSS Statistics, v20.0; IBM Corp). Two-way analysis of variance (ANOVA) and Bonferroni multiple comparison tests were used to compare the inlay preparation designs of the teeth between the experimental groups (α = 0.05).

## Results

Table 2 shows the statistical results of the two-way ANOVA for the mean deviation of trueness, mean maximum positive and negative deviations, and mean deviation of precision for each variable. The trueness was significantly affected by both the width and depth (*p* < 0.05), whereas the mean maximum positive deviation was affected by width (*p* < 0.05). The mean maximum negative deviation was significantly affected by all three factors (*p* < 0.05). In contrast, the precision was significantly affected by the depth and the interaction between the depth of the occlusal cavity and the width of the gingival floor (*p* < 0.05).

**Table 2:** Results of 2-way ANOVA of parameters

Parameter	Source	df	SS	MS	F	*p*
**Average deviation for trueness**	Width	1	13.94	13.94	6.34	0.016
	Depth	1	11.98	11.98	5.45	0.025
	Width × depth	1	0.38	0.38	0.17	0.679
**Mean maximum positive deviation**	Width	1	1612.90	1612.90	32.73	0
	Depth	1	8.10	8.10	0.16	0.688
	Width × depth	1	25.60	25.60	0.52	0.476
**Mean maximum negative deviation**	Width	1	11767.90	11767.90	130.41	0
	Depth	1	577.60	577.60	6.40	0.016
	Width × depth	1	20160.10	20160.10	223.46	0
**Average deviation for precision**	Width	1	0.92	0.92	0.48	0.489
	Depth	1	32.60	32.60	17.03	0
	Width × depth	1	9.90	9.90	5.17	0.024

*df* Degrees of freedom, *MS* Mean squares, *SS* Sum of squares

**Fig. 3:**
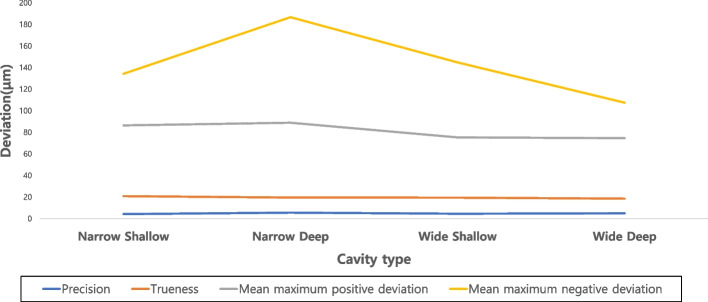
Overall average deviation for trueness, maximum deviations, and precision in tested groups of cavity types

Table 3 and Fig. 3 show the results for each variable in each cavity type group. As per the cavity type, the average deviation for trueness ranged from 18.56 ± 1.48 μm to 20.84 ± 1.16 μm. The narrow shallow group had significantly higher values than the wide deep group (*p* < 0.05), while there was no significant difference between the narrow deep and wide shallow groups, respectively (*p* > 0.05). The wide deep group had the lowest average deviation value for trueness and there were no significant difference between the narrow deep and wide shallow groups (*p* > 0.05). The mean maximum positive deviation value ranged from 74.6 ± 4.81 μm to 88.9 ± 7.20 μm, and the mean maximum negative deviation value ranged from 107.5 ± 16.24 μm to 186 ± 6.17 μm. The mean maximum positive and negative values were the lowest in the wide-deep group. For the mean maximum positive deviation, the wide groups had significantly lower values than the narrow groups (*p* < 0.05). The average deviation for precision ranged from 4.24 ± 0.79 μm to 5.56 ± 1.96 μm. The narrow shallow group had a lowest value and narrow deep group had highest value.

**Table 3:** Mean ± standard deviation (µm) values of parameters of all experimental groups

Cavity	Average deviation for trueness	Mean maximum positive deviation	Mean maximum negative deviation	Average deviation for precision
Narrow shallow	20.84 ± 1.16^A^	86.40 ± 9.81 ^A^	134.20 ± 5.41 ^A^	4.24 ± 0.79 ^A^
Narrow deep	19.55 ± 1.47^AB^	88.90 ± 7.20 ^A^	186.7 ± 6.17 ^B^	5.56 ± 1.96 ^B^
Wide shallow	19.46 ± 1.76^AB^	75.30 ± 5.08 ^B^	144.8 ± 5.47 ^A^	4.57 ± 1.39 ^AC^
Wide deep	18.56 ± 1.48^B^	74.60 ± 4.81^B^	107.5 ± 16.2 4 ^C^	4.95 ± 1.12 ^BC^

Different superscript letters within the same column indicate statistical differences between cavity types by Bonferroni multiple comparison test (*p* < 0.05)

**Fig. 4:**
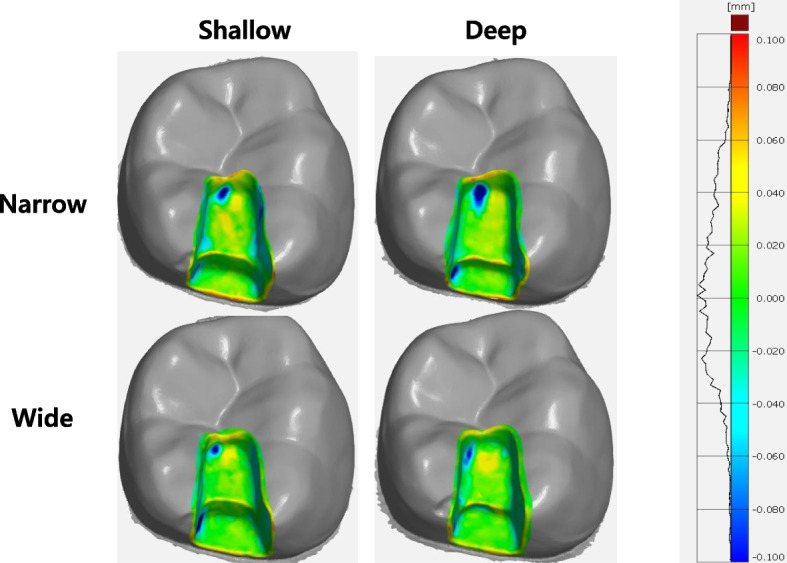
Qualitative analysis of trueness in tested groups of cavity types

Figure 4 shows the pattern of the mean maximum positive deviation and the mean maximum negative deviation in the color-coded map overlapped with the reference data. In most groups, the most positive deviations were mainly observed in the gingival margin of the proximal box. Additionally, a positive deviation was observed at the line where the occlusal floor met the pulpal wall of the proximal box. In contrast, a negative deviation was observed at the internal point angle of the occlusal cavity.

## Discussion

The aim of our study was to evaluate the influence of the depth of the occlusal cavity and width of the gingival floor of the proximal box on the trueness and precision of digital IOS for inlay cavities. The highest average deviation for trueness was observed in the narrow-shallow group and the lowest in the wide-deep group. In contrast, for precision, the narrow-shallow group showed the lowest average deviation, and shallow groups showed lower values than deep groups. Therefore, the null hypothesis that the depth of the occlusal cavity and width of the gingival floor of the proximal box would not affect the accuracy of the digital scan data was rejected.

In our study, the average deviation for trueness ranged from 18.56 ± 1.48 μm to 20.84 ± 1.16 μm. The obtained parameter values in the experimental groups should be carefully interpreted. A low average deviation represents high trueness or precision of the scan data obtained from the IOS. The deviation value of trueness decreased as the width of the gingival floor of the proximal box increased and the depth of the occlusal cavity deepened. Generally, the IOS can be classified according to the data capture principle into active triangulation, confocal microscopy, optical coherence tomography, and active wavefront sampling [7, 15]. Particularly, Primescan, which was used in our study, is based on both optical triangulation and confocal microscopy [16]. Additionally, it works on the optical measurement principle based on the shortwave length with optical high-frequency contrast analysis for dynamic depth scans and high-resolution sensors [17]. Generally, the deviation of the digital scan can be minimized when the IOS camera is positioned perpendicular to the surface to be scanned, the light is reflected directly at the surface, and the magnitude of the deviation increases as the camera moves away from the vertical plane [15]. The direction of the light from the IOS must be parallel to the long axis of the teeth when creating the digital scanning image, without touching the cavity wall. If there is an obstacle, the light will not reflect certain components of the cavity surface, which results in a partial loss of the impression [18]. As the surface area increased in the preparation designs, the amount of the light from the IOS reaching the inner surface of the cavity increased. In our study, the highest average deviation for trueness was observed in the narrow-shallow group and the lowest in the wide-deep group. The result of the two-way ANOVA consistently indicated that the width and depth affected trueness.

Positive and negative deviations are related to clinically relevant parameters. A positive deviation results in a thinner restoration, increasing the risk of fracture and leaving a large interfacial discrepancy. This space would be filled with a thicker layer of cement, which can compromise restoration retention. In addition, the greater shrinkage stress generated during polymerization due to the increased amount of resin cement may cause debonding at the interface between the tooth and cement. Interfacial debonding may cause postoperative sensitivity (especially upon mastication), marginal discoloration, and secondary caries. In contrast, a negative deviation would induce ill-fitting and premature contact with the opposing tooth. For this reason, more time would likely be spent on occlusal adjustment of the restoration [7, 14]. In our study, the mean maximum positive deviation value ranged from 74.6 ± 4.81 μm to 88.9 ± 7.20 μm, and the mean maximum negative deviation value ranged from 107.5 ± 16.24 μm to 186 ± 6.17 μm. The mean maximum positive and negative deviations were the lowest in the wide-deep group. In the present experiment, the mean maximum negative deviation was less than the positive deviation in all the groups. As shown in the color-coded map, the most positive deviations were mainly observed in the gingival margin of the proximal box. Additionally, a positive deviation was observed at the line where the occlusal floor met the pulpal wall of the proximal box. In contrast, a negative deviation was observed at the internal point angle of the occlusal cavity. This is consistent with the findings of a previous study that reported deviations in areas of a sudden change in curvature within the cavity [4].

Clinically, the IOS requires a range of single small-sized scans with a rendering motion for acquiring digital scanning images and subsequent superimpositioning to generate a full-sized surface image. The IOS must have an adequate depth of field (DOF) to obtain accurate images. Confocal imaging projects laser light onto a target object through a filtering pinhole. It has a high scan accuracy because it removes the out-of-focus light from the reflected light. However, when it is outside the DOF range, the out-of-focus signal decreases and the noise increases, leading to image blur [19]. The Primescan IOS used in this study had a DOF of approximately 20 mm, which is greater than that of the previous Omnicam (CEREC Omnicam, Dentsply Sirona) scanner model [4]. This is advantageous in reproducing the sharpness of the edges. However, if the surface of the acquired image is out of the DOF range, the image accuracy may be negatively affected. Overall accuracy is altered depending on the quality of the single scans, matching algorithm for superimposition, and object size and distance from the IOS. Default superimposition may further accumulate, leading to deformation and changes in the dimension and shape of the datasets [15]. In the present study, the average deviation for precision value ranged from 4.24 ± 0.79 μm to 5.56 ± 1.96 μm, with low deviation values. The shallow groups had lower deviation precision values than the deep groups, and the narrow-deep group had the highest value. When obtaining the optical scan, a deeper occlusal cavity may hinder access to light from the scanner when the IOS is not suitably positioned. Additionally, the precision values were lower than the trueness values, ranging from 18.56 ± 1.48 μm to 20.84 ± 1.16 μm for all test groups. This means that the digital scan data are not considerably affected by repeated scanning with the Primescan IOS.

Our study had several limitations. First, real human teeth and an industrial scanner for the reference scanner were not used. Natural human enamel possesses optical properties, such as reflection and dispersion, which are different from those of artificial resin teeth. In addition, the study was performed in vitro, outside the oral environment. The oral cavity includes saliva and blood, and there are several related challenges, such as instability in scanner placement during the scanning procedure, that we could not assess as a result. Clinical studies on factors that determine the accuracy of other cavity types for CAD/CAM restorations when using diverse types of IOS should be conducted.

## Conclusions

With the limitations of the present in vitro study, we can conclude that the design of different inlay cavity configurations affected the accuracy of the digital IOS. The highest average deviation for trueness was observed in the narrow-shallow group and the lowest in the wide-deep group. In contrast, for the precision, the narrow-shallow group showed the lowest average deviation, and the shallow groups showed lower values than the deep groups.

## Data Availability

The STL files and 3D surface models obtained in this study with the IOS as well as the reference files obtained with the laboratory scanner belong to School of Dentistry, Pusan National University property, and are therefore available only upon reasonable request, after approval by the University. The datasets used and/or analyzed during the current study are available from the corresponding author on reasonable request.

## Abbreviations

CAD: Computer-assisted-design
CAM: Computer-assisted-manufacturing
IOS: Intraoral scanner
STL: Standard tessellation language
DOF: Depth of field
